# Supplementary material for: Layer Hall effect induced by hidden Berry curvature in antiferromagnetic insulators
Source: Natl Sci Rev. 2022 Aug 6;11(2):nwac140. doi: 10.1093/nsr/nwac140 (PMC10804226; doi:10.1093/nsr/nwac140)
Supplement: nwac140_Supplemental_File [file nwac140_supplemental_file.pdf]

Supplementary Material

Rui Chen, Hai-Peng Sun, Mingqiang Gu, Chun-Bo Hua, Qihang Liu, Hai-Zhou Lu, and X. C. Xie

CONTENTS

SI. Model, method, and symmetry S1

SII. Using perpendicular electric field to reveal Layer Hall effect S2

SIII. Extracting the half-quantization in the axion insulator S9

SIV. Measurement proposals for layer Hall effect S10
A. Nonlocal measurement S10
B. Hall measurement S12

SV. DFT calculations S13

References S15

SI. MODEL, METHOD, AND SYMMETRY

We start with an effective $k \cdot p$ model for the antiferromagnetic topological insulator around the Γ point [S1–S4],

$$\mathcal{H}(\mathbf{k}) = H_0 + H_X(z) + V(z), \tag{S1}$$

with $H_0(\mathbf{k}) = \sum_{i=4}^{4} d_i(\mathbf{k})\Gamma_i$, $d_1 = A_1 k_x$, $d_2 = A_1 k_y$, $d_3 = A_2 k_z$, and $d_4 = M_0 - B_1 k_z^2 - B_2\left(k_x^2 + k_y^2\right)$. $\Gamma_{j=1,2,3} = s_i\sigma_1$, $\Gamma_4 = s_0\sigma_3$, and σ and τ are Pauli matrices for the spin and orbital subspaces, respectively. C_0, D_i, M_0, B_i, and A_i are model parameters, where $i = 1, 2$. H_0 respects time-reversal symmetry $\mathcal{T} = is_2\sigma_0\mathcal{K}$ and inversion symmetry $\mathcal{P} = \Gamma_4$. For $M_0 > 0$, H_0 describes a time-reversal invariant topological insulator around the Γ point, and hosts a single Dirac cone on each surface. For $M_0 < 0$, H_0 describes a normal insulator. We take into account the spatial profile of the exchange field $H_X(z)$ in the antiferromagnetic order. For simplicity, we consider the exchange field $H_X = -m(z)s_z\sigma_0$. Moreover, we assume the perpendicular electric field E_z produces a potential $V(z)$, which is an odd function of z.

We discretize $\mathcal{H}(\mathbf{k})$ on a cubic lattice as

$$H = \sum_{\mathbf{r}} \phi_{\mathbf{r}}^{\dagger} V_{\mathbf{r}} \phi_{\mathbf{r}} + \left(\sum_{\mathbf{r},\alpha=x,y,z} \phi_{\mathbf{r}}^{\dagger} T_{\alpha} \phi_{\mathbf{r}+\delta\hat{\alpha}} + \text{ H.c.} \right), \tag{S2}$$

with $V_{\mathbf{r}} = \Gamma_4\left(M_0 - 2B_1 - 4B_2\right) + m(-1)^z s_z\sigma_0 + V\left[z - (n_z+1)/2\right] s_0\sigma_0$, $T_x = -iA_2\Gamma_1/2 + B_2\Gamma_4$, $T_y = -iA_2\Gamma_2/2 + B_2\Gamma_4$, and $T_z = -iA_1\Gamma_3/2 + B_1\Gamma_4$. m describes the amplitude of the intra-layer ferromagnetic order, and V measures the potential on each layer induced by the perpendicular electric field E_z. For $m \neq 0$ and $V = 0$, the system in Eq. (S2) describes a trivial insulator when $M_0 < 0$, and an antiferromagnetic insulator when $M_0 > 0$.

The BZ-integrated hidden Berry curvature localized on each layer is obtained through the noncommutative real-space Kubo formula [S5–S8]

$$\Omega_n(z) = 2\pi i \,\mathrm{Tr}\left\{\hat{P}\left[-i\left[\hat{x}, \hat{P}\right], -i\left[\hat{y}, \hat{P}\right]\right]\right\}_z, \tag{S3}$$

with periodic boundary conditions along the x and y directions. Here, $\hat{x}$ and $\hat{y}$ are the coordinate operators and $\mathrm{Tr}\{\cdots\}_z$ is the trace over the z-th layer ($z = 1, 2, 3, ..., n_z$). $\hat{P}$ is the projector onto the occupied states of H. Eq. (S3) is recognized as a local Chern marker representing the real-space projected Chern number in the z-direction [S9, S10]. The layer-resolved Hall conductance is $\sigma_{xy}(z) = \frac{e^2}{h}\Omega_n(z)$, and the total Hall conductance is given by $\sigma_{xy} = \sum_z \sigma_{xy}(z)$.

Table S2 illustrates the symmetries of the tight-binding model of the antiferromagnetic films. For odd-layer films, the system shows anomalous Hall effect due to the broken of the $\mathcal{PT}$ symmetry.

	$\mathcal{T}$	$\mathcal{P}$	$\mathcal{PT}$	Hall	Layer Hall
NM	✓	✓	✓	×	×
AFM	×	×	✓	×	✓
AFM with E_z field	×	×	×	✓	✓

Tab. S1. Symmetries of the even-layer AFM films. NM stands for nonmagnetic. For an AFM insulator, the net Hall conductance σ_{xy} is zero due to the global $\mathcal{PT}$ symmetry. Each layer breaks the $\mathcal{PT}$ symmetry locally, which allows nonvanishing local Hall conductance and establishes the layer Hall effect. In the presence of the perpendicular electric field E_z, the nonvanishing anomalous Hall conductance is not simply originated from the $\mathcal{PT}$ breaking, but the emergence of the layer-dependent Berry curvature in the layer Hall effect. The role of the perpendicular electric field is just to separate the hidden Berry curvature of different layers compensated by the global $\mathcal{PT}$ symmetry.

Odd layer	$\mathcal{T}$	$\mathcal{P}$	$\mathcal{PT}$	Hall	Layer Hall
Nonmagnetic	✓	✓	✓	×	×
AFM	×	✓	×	✓	×
AFM with E_z field	×	×	×	✓	×

Tab. S2. Symmetries of the odd-layer antiferromagnetic films.

SII. USING PERPENDICULAR ELECTRIC FIELD TO REVEAL LAYER HALL EFFECT

In the bilayer system, the hidden Berry curvatures localized on each layer have degenerate energy due to the $\mathcal{PT}$ symmetry. In this section, we show how the layer-locked Berry curvature and the layer Hall effect become observable by applying a perpendicular electric field E_z. As shown in Fig. S1(a), E_z breaks the $\mathcal{PT}$ symmetry and induces an energy offset between the states of the two layers. When the Fermi energy cuts the valence band of the top layer, the system is dominated by the occupied bands with the negative Berry curvature contributed by the bottom layer. As a result, a net anomalous Hall conductance appears. Here, we emphasize that the net anomalous Hall conductance is not simply originated from the $\mathcal{PT}$ breaking, but the emergence of the hidden Berry curvature. The role of E_z is predominately to separate the hidden Berry curvature of different layers compensated by the global $\mathcal{PT}$ symmetry. This can be easily verified by that the Hall conductance of an AFM topological insulator is much larger than that of a trivial insulator under the same electric field [see Fig. S1(a) and Tab. S2].

The above scenarios can be observed more clearly in Figs. S1(b-g). In the presence of the perpendicular electric field, the surface bands split. As indicated by the arrows shown in Fig. S1(a), the perpendicular electric field shifts the surfaces states located at opposite surfaces to opposite directions in energy. For a moderate electric-field strength, the two surface valence bands below (or above) the band gap are well separated. The systems are characterized by nonzero anomalous Hall conductances when the Fermi energy is located in the colored regions.

Below, we show more results similar to those in Fig. S1, but for different numbers of layers, ranging from $n_z = 3$ to $n_z = 8$. For different even-layer films, the results are similar to the case with $n_z = 2$. The odd-layer films have no $\mathcal{PT}$ symmetry, and the layer Hall effect is absent in these systems. We also show the results for different system sizes $n_{x,y}$. The Hall conductances converge as $n_{x,y}$ increase.

Figure S2 shows $\sigma_{xy}(z)$ as functions of m for different system size. It is found that $\sigma_{xy}(z)$ converges as the system size increases. The finite size effect is eliminated for system large enough such as $n_x = n_y = 30$.

For the odd-layer films with $n_z = 3, 5$ [Fig. S3], the results are different from the case of $n_z = 2$. In the absence of the perpendicular electric field, the film has no $\mathcal{PT}$ symmetry and hosts a Chern insulator characterized by $\sigma_{xy} = e^2/h$, contributed by two $e^2/2h$ from each of the top and bottom surface [Fig. S3]. We notice that the layer Hall effect is absent in such a system. When the electric field is introduced, the quantized Hall conductance plateau splits into two minor plateaus since the two surfaces are decoupled in energy. The two minor plateaus approaches to half-quantized value $\sigma_{xy} \approx e^2/2h$ when the two surfaces are fully-decoupled in energy [Fig. S3(c)]. Moreover, in contrast to the case of $n_z = 2$ or $n_z = 4$, we observe that the chirality of the induced Hall effect cannot be tuned by modulating the electric-field direction [Fig. S3(c)].

Figures S4-S9 show the spectra and corresponding Berry curvature distributions and wave function distributions for $n_z = 2-7$. For even layers such as $n_z = 4$ [Fig. S6], the results are similar to the case of $n_z = 2$ illustrated in the main text. For odd layers, in the absence of the perpendicular electric field ($V = 0$), the two surfaces are characterized by the same Berry curvature because the two surfaces have the same magnetization. When the Fermi energy is located in the energy gap ($E_F = 0$), the system corresponds to a Chern insulator, characterized by a quantized Chern number $C = 1$ and contributed by the surface states of the two surfaces. In the presence of the perpendicular electric

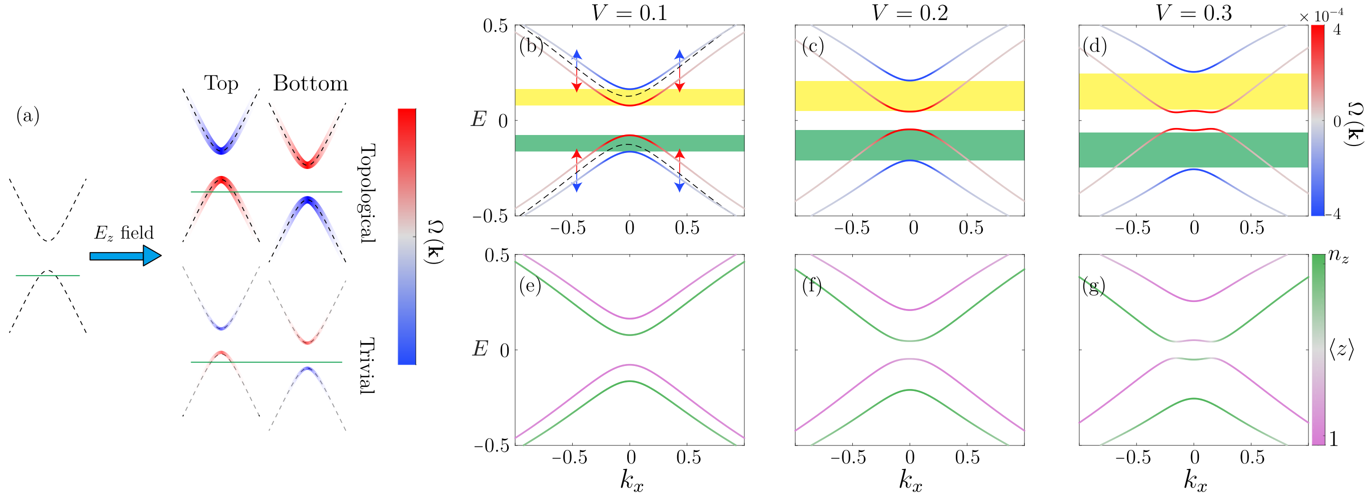

Fig. S1. (a) Schematic of the layer Hall effect with a perpendicular electric field. The perpendicular electric field induces a potential difference between the top and bottom surfaces, leading to an imbalance of their Hall conductances, as a result of the uncompensated hidden Berry curvature of different layers when the global $\mathcal{PT}$ symmetry is broken. (b, e) Energy spectra with $n_z = 2$ for $V = 0.1$. The color bars correspond to the Berry curvature distributions $\Omega(k_x, k_y)$ in (b) and wave function distributions in (e). (c, f) and (d, g) are the same as (b, e), except that $V = 0.2$ and $V = 0.3$, respectively. In (b-d), there appear anomalous Hall effects with opposite chiralities when the Fermi energy is located in the yellow and green regions. The dashed lines in (b) correspond to $V = 0$, and the red and blue arrows indicate the shifting tendency of the energy bands in response to the perpendicular electric field.

field, the surface bands split. As indicated by the arrows shown in Figs. S5(a), S7(a), and S9(a), the perpendicular electric field shifts the surfaces states located at opposite surfaces to opposite directions in energy. For a moderate electric-field strength, the two surface valence bands below (or above) the band gap are well separated. The systems are characterized by nearly half-quantized conductances when the Fermi energy is located in the colored regions.

Figure S10 shows the Hall conductance as functions of V and E_F for $n_z \in [3, 8]$. The perpendicular electric field can induce the anomalous Hall effect for different even-layer films, and split the quantized Hall conductance to two minor regions for different odd-layer films.

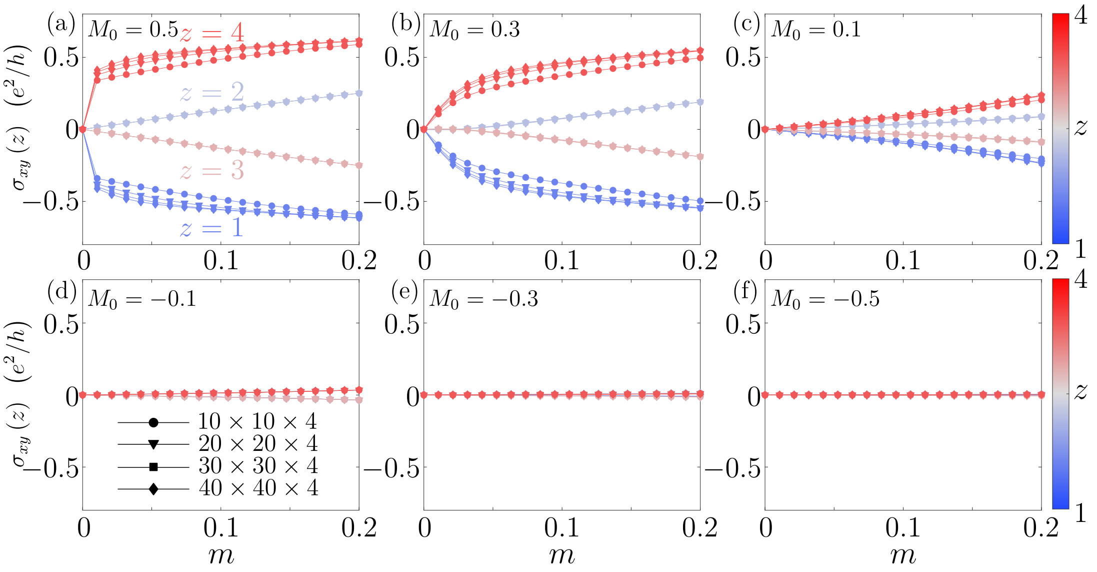

Fig. S2. The numerically calculated Hall conductances $\sigma_{xy}(z)$ as functions of m for different M_0 and system sizes. Here, the color distinguishes different z and the system size is $n_z = 4$ with $n_x = n_y = 10$ (circle), $n_x = n_y = 20$ (triangle), $n_x = n_y = 30$ (square), and $n_x = n_y = 40$ (diamond).

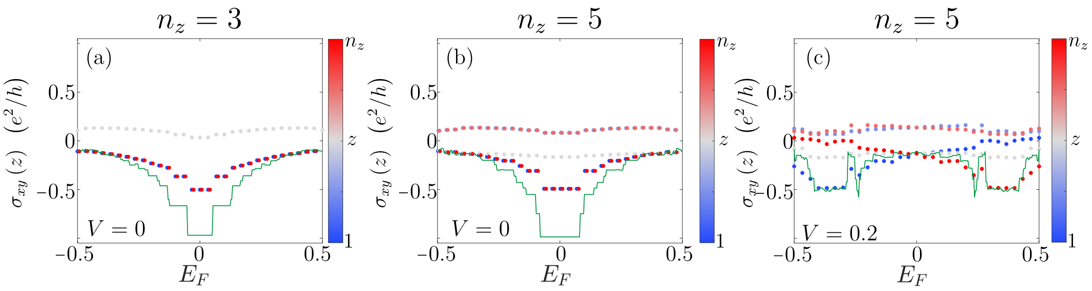

Fig. S3. (a-c) The numerically calculated Hall conductances as a function of the Fermi energy E_F with (a) $n_z = 3$, $V = 0$, (b) $n_z = 5$, $V = 0$, and (c) $n_z = 5$, $V = 0.2$, respectively. In (c), the disorder strength is $U = 1$.

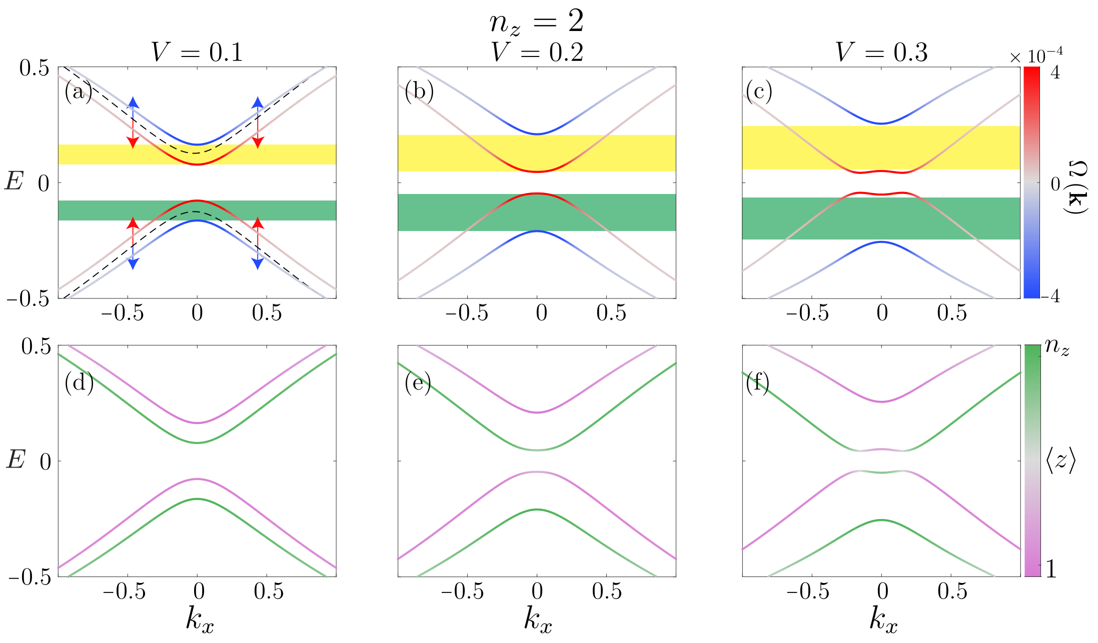

Fig. S4. (a, d) Energy spectra with $n_z = 2$ for $V = 0.1$. The color bars correspond to the Berry curvature distributions $\Omega(k_x, k_y)$ in (a) and wave function distributions in (d). (b, e) and (c, f) are the same as (a, d), except that $V = 0.2$ and $V = 0.3$, respectively. In (a-c), there appear anomalous Hall effects with opposite chiralities when the Fermi energy is located in the yellow and green regions.

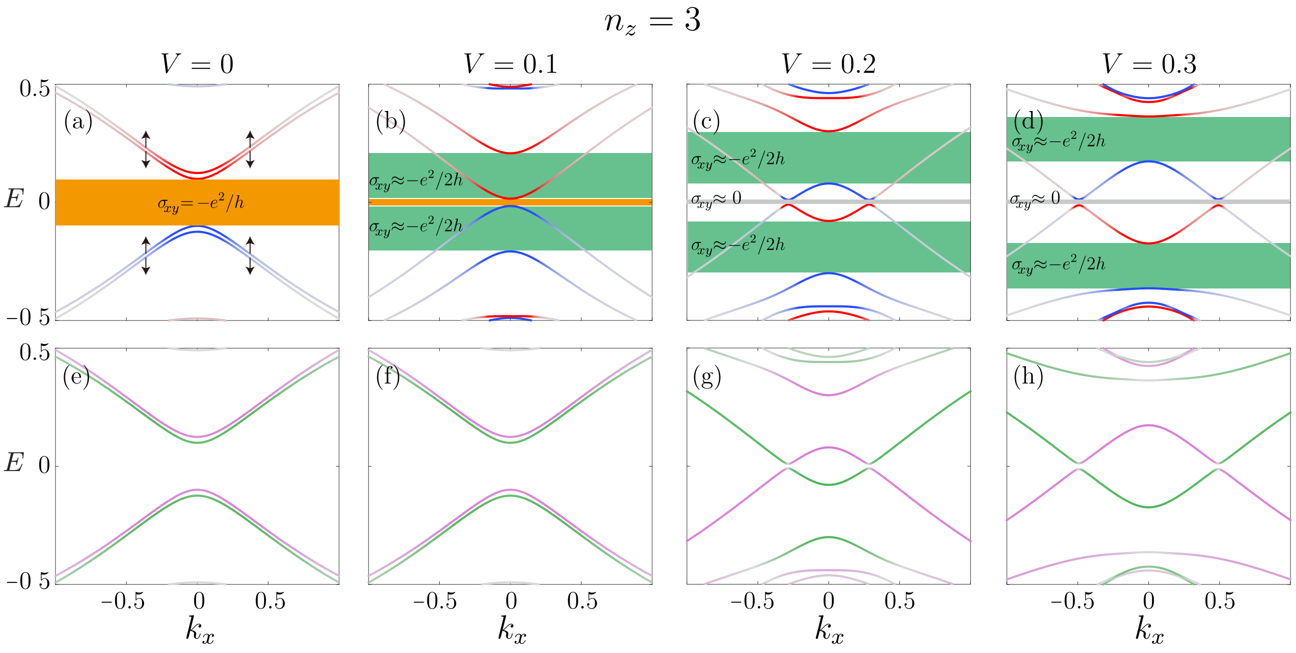

Fig. S5. (a, e) Energy spectra with $n_z = 3$ for $V = 0$. The color bars correspond to the Berry curvature distributions $\Omega(k_x, k_y)$ in (a) and wave function distributions in (e). (b, f), (c, g), and (d, h) are the same as (a, e), except that $V = 0.1$, $V = 0.2$, and $V = 0.3$, respectively. In (a-d), the Hall conductance σ_{xy} is nearly $-e^2/h$, 0, and $-e^2/2h$ when the Fermi energy is located in the orange, gray, and green regions, respectively.

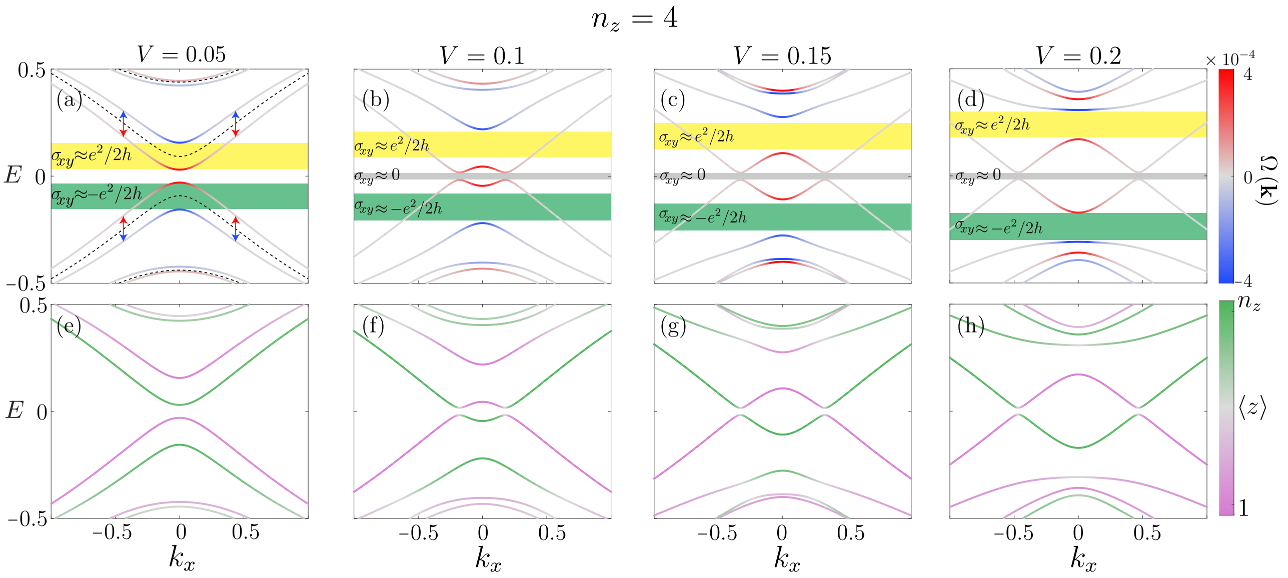

Fig. S6. (a, d) Energy spectra with $n_z = 4$ for $V = 0.1$. The color bars correspond to the Berry curvature distributions $\Omega(k_x, k_y)$ in (a) and wave function distributions in (d). (b, e) and (c, f) are the same as (a, d), except that $V = 0.15$ and $V = 0.2$, respectively. In (a-c), the Hall conductance σ_{xy} is nearly $e^2/2h$, 0, and $-e^2/2h$ when the Fermi energy is located in the yellow, gray, and green regions, respectively.

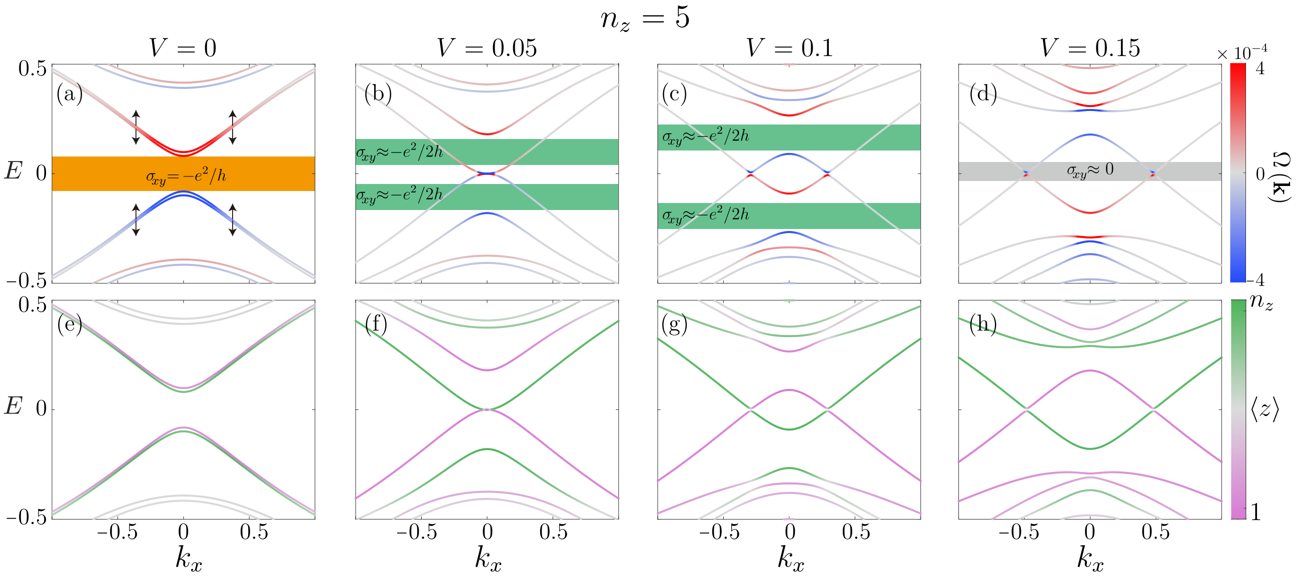

Fig. S7. (a, e) Energy spectra with $n_z = 5$ for $V = 0$. The color bars correspond to the Berry curvature distributions $\Omega(k_x, k_y)$ in (a) and wave function distributions in (e). (b, f), (c, g), and (d, h) are the same as (a, e), except that $V = 0.05$, $V = 0.1$, and $V = 0.15$, respectively. In (a-d), the Hall conductance σ_{xy} is nearly $-e^2/h$, 0, and $-e^2/2h$ when the Fermi energy is located in the orange, gray, and green regions, respectively.

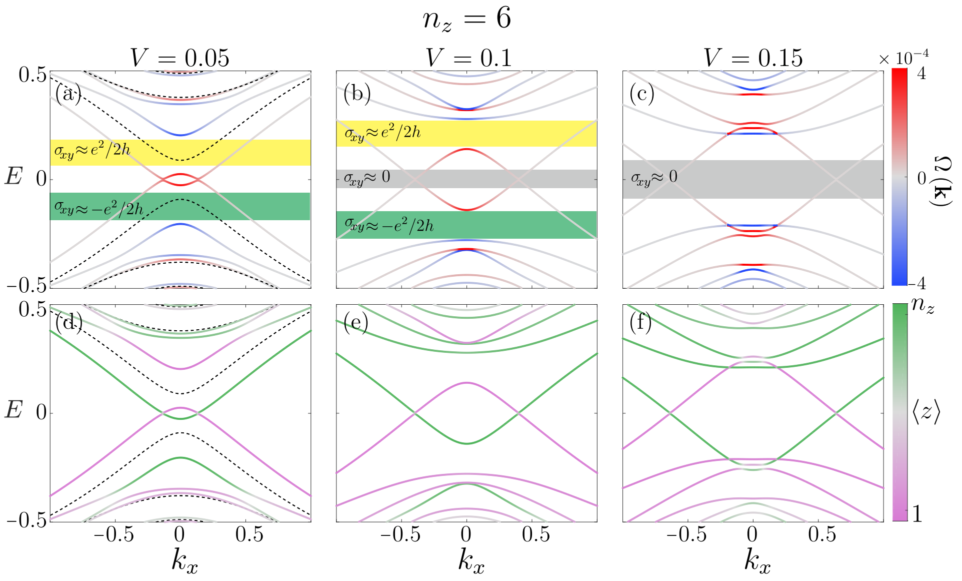

Fig. S8. (a, d) Energy spectra with $n_z = 6$ for $V = 0.0.05$. The color bars correspond to the Berry curvature distributions $\Omega(k_x, k_y)$ in (a) and wave function distributions in (d). (b, e) and (c, f) are the same as (a, d), except that $V = 0.1$ and $V = 0.15$, respectively. In (a-c), the Hall conductance σ_{xy} is nearly $e^2/2h$, 0, and $-e^2/2h$ when the Fermi energy is located in the yellow, gray, and green regions, respectively.

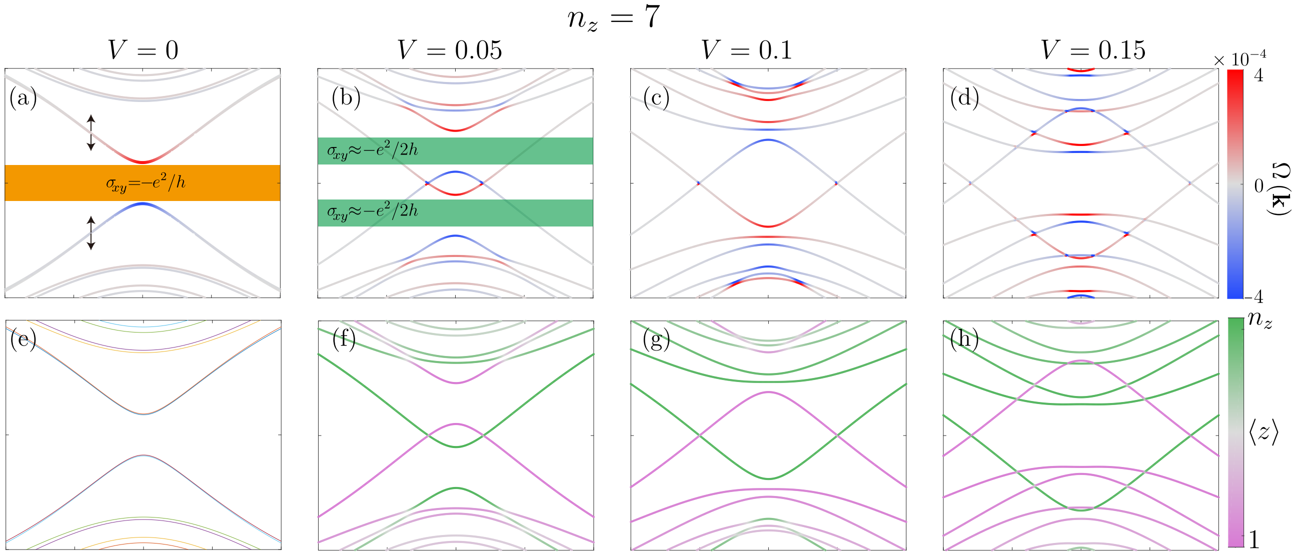

Fig. S9. (a, e) Energy spectra with $n_z = 7$ for $V = 0$. The color bars correspond to the Berry curvature distributions $\Omega(k_x, k_y)$ in (a) and wave function distributions in (e). (b, f), (c, g), and (d, h) are the same as (a, e), except that $V = 0.05$, $V = 0.1$, and $V = 0.15$, respectively. In (a-d), the Hall conductance σ_{xy} is nearly $-e^2/h$, 0, and $-e^2/2h$ when the Fermi energy is located in the orange, gray, and green regions, respectively.

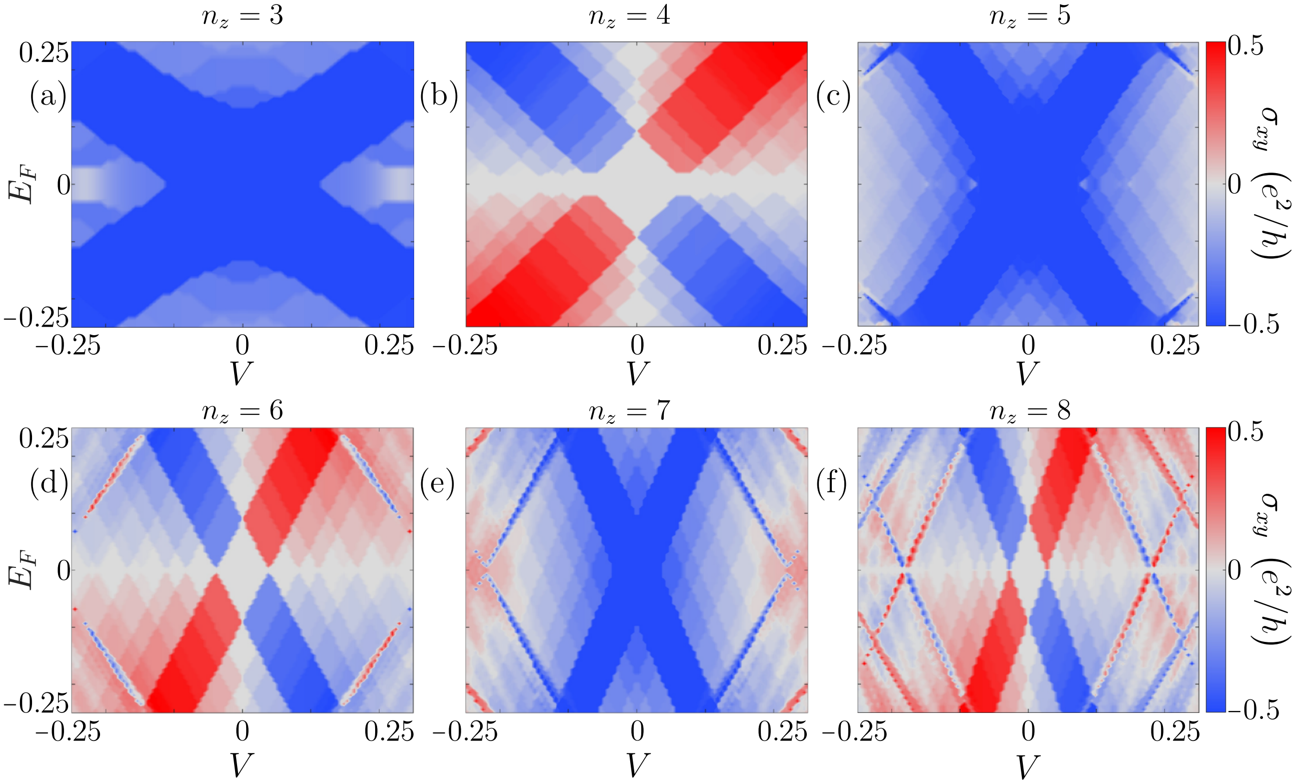

Fig. S10. Numerically calculated σ_{xy} as a function of V and E_F, with $n_z = 3 - 8$.

SIII. EXTRACTING THE HALF-QUANTIZATION IN THE AXION INSULATOR

Figure S11(a) shows the numerically calculated Hall conductance $\sigma_{xy}(z)$ as a function of layer index z for different m with $n_z = 4$, where m describes the amplitude of the intra-layer ferromagnetic order. The layer Hall effect is enhanced near the two surfaces because it is contributed by both the hidden Berry curvature induced anomalous Hall effect and the half-quantized surface Hall effect. To extract the half-quantization and suppress the oscillatory behavior of the bulk layers, we then define the renormalized Hall conductance [S11] as

$$\tilde{\sigma}(z) = \frac{\sigma(z-1) + \sigma(z)}{2}, z = 2, \cdots, n_z, \tag{S4}$$

with $\tilde{\sigma}(1) = \sigma(1)/2$ and $\tilde{\sigma}(n_z+1) = \sigma(n_z)/2$. In this way, each layer is counted once except for the layer at the surfaces, which is counted with weight 1/2. This can be regarded as an application of the sliding window averaging method [S11].

Figure S11(b) shows the numerically calculated renormalized Hall conductance $\tilde{\sigma}_{xy}(z)$ as a function of layer index z for different m with $n_z = 4$. The surface Hall effect is extracted from the hidden Berry curvature induced anomalous Hall effect. We then define $\tilde{\sigma}^t_{xy} = \tilde{\sigma}(1) + \tilde{\sigma}(2)$ and $\tilde{\sigma}^b_{xy} = \tilde{\sigma}(n_z) + \tilde{\sigma}(n_z+1)$, which correspond to the contributions from the surface Hall effect. As shown in Fig. S11(c), $\tilde{\sigma}^t_{xy}$ approaches a half-quantized value for m is large enough. Furthermore, this phenomenon can be observed more clearly for the case of $n_z = 6$ shown in Figs. S11(d-f). Therefore, we show that the half-quantization can be extracted from the layer Hall effect in the axion insulator.

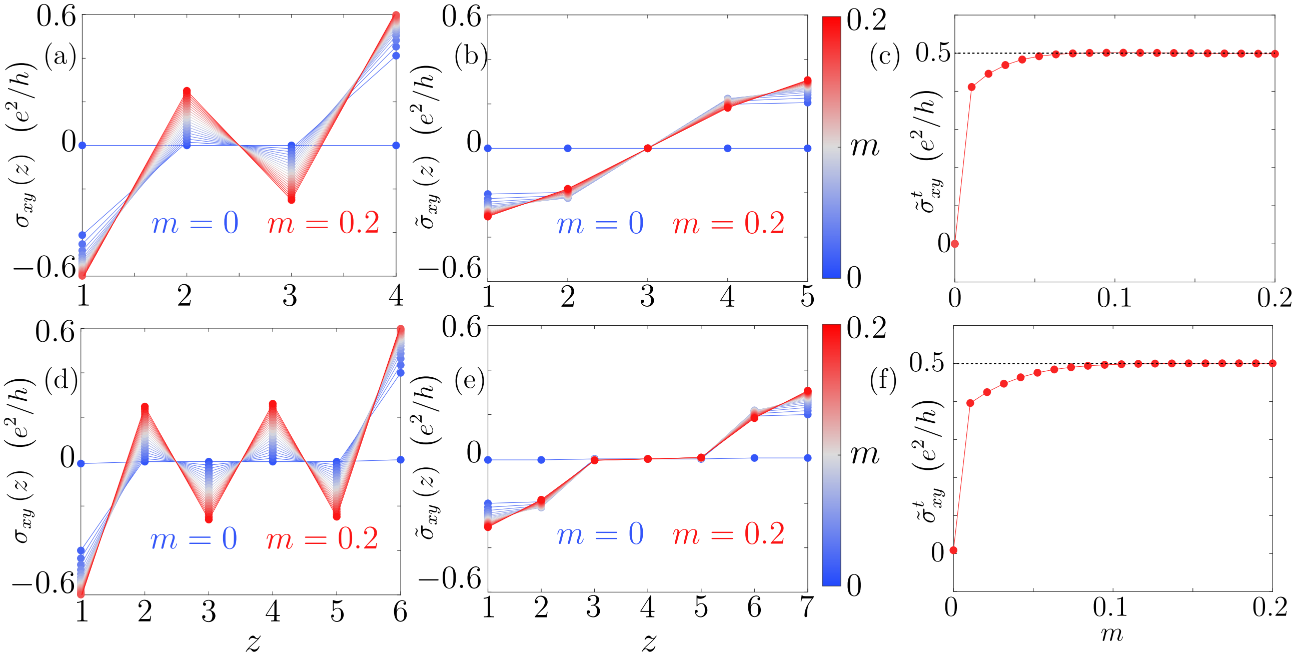

Fig. S11. Numerically calculated (a) Hall conductance $\sigma_{xy}(z)$ and (b) renormalized Hall conductance $\tilde{\sigma}_{xy}(z)$ as functions of z for different m. Here, the color distinguishes different m. (c) Numerically calculated renormalized Hall conductance $\tilde{\sigma}^t_{xy}$ as a function of m. In (a-c), the system size is $n_z = 4$ and $n_x = n_y = 40$. (d-f) The same as (a-c), except for $n_z = 6$.

SIV. MEASUREMENT PROPOSALS FOR LAYER HALL EFFECT

To measure the layer Hall effect, one can also use the nonlocal measurement without applying a perpendicular electric field , besides the standard six-terminal Hall-bar measurement in the presence of a perpendicular electric field. We will illustrate these two measurements as follows. The two measurements can be simulated by using the Landauer-Büttiker formula [S12–S14] and the recursive Green's function method [S15, S16]. The current flowing into terminal p is given by

$$I_p = \frac{e^2}{h} \sum_{q \neq p} T_{pq} \left(E_f\right) \left(V_p - V_q\right). \tag{S5}$$

$T_{pq}\left(E_F\right) = \mathrm{Tr}\left[\Gamma_p G^r \Gamma_q G^a\right]$ is the transmission coefficient from terminal q to terminal p at the Fermi energy E_F with $p \neq q$. The linewidth function $\Gamma_p(E_F) = i\left[\Sigma_p^r - \Sigma_p^a\right]$ and the Green's functions $G^{r/a}(E_F)$ are calculated from $G^r(E_F) = \left[G^a(E_F)\right]^\dagger = \left[E_F I - H_C - \sum_p \Sigma_p^r\right]^{-1}$, where H_C is the Hamiltonian matrix of the AFM insulator and $\Sigma_p^{r/a}$ are the retarded/advanced self-energies of the electrodes.

A. Nonlocal measurement

The quantum spin Hall effect is characterized by quantized counter-propagating edge channels, which gives rise to the nonlocal transport as a smoking gun evidence [S17]. Analogous to the quantum spin Hall effect, the layer Hall effect is characterized by non-zero and non-half-quantized counter-propagating edge channels in thinfilms, and nearly half-quantized counter-propagating edge channels in the axion-insulator phase. We expect that the counter-propagating currents in the layer Hall effect can be detected through the nonlocal measurements. However, unlike in the quantum spin Hall effect, the counter-propagating currents of the AFM topological insulator films can be contributed by both the layer Hall effect and the side surface Dirac cone. Therefore, we need to extract the layer Hall effect from the side-surface contribution in the nonlocal measurement.

To serve this purpose, we investigate the nonlocal measurements [S18] in a four-terminal device as depicted in Figs. S12(a-b). We choose four identical electrodes. The transmission matrix is found to have the form

$$T = \begin{pmatrix} 0 & T_1 & T_3 & T_2 \\ T_2 & 0 & T_1 & T_3 \\ T_3 & T_2 & 0 & T_1 \\ T_1 & T_3 & T_2 & 0 \end{pmatrix}, \tag{S6}$$

where T_1 and T_2 are the clockwise and counter-clockwise transmission coefficients between two neighboring terminals, respectively, and T_3 is the transmission coefficient between two non-neighboring terminals, as shown in Figs. S12(a-b). Our numerical calculations reveal that $T_3 = 0$, showing the dominance of the edge transport.

Specifically, the above equation in Eq. (S5) can be written in a matrix form

$$\begin{pmatrix} I_1 \\ I_2 \\ I_3 \\ I_4 \end{pmatrix} = \frac{e^2}{h} \begin{pmatrix} \sum_{p=1}^3 T_i & -T_1 & -T_3 & -T_2 \\ -T_2 & \sum_{p=1}^3 T_i & -T_1 & -T_3 \\ -T_3 & -T_2 & \sum_{p=1}^3 T_i & -T_1 \\ -T_1 & -T_3 & -T_2 & \sum_{p=1}^3 T_i \end{pmatrix} \begin{pmatrix} V_1 \\ V_2 \\ V_3 \\ V_4 \end{pmatrix}. \tag{S7}$$

We apply a bias across terminal 1 and terminal 2, and the currents in the voltage probes (terminals 3 and 4) are set to zero. Owing to the current conservation, $I_1 = -I_2 = I$. We set $V_4 = 0$, which allows us to truncate the fourth row and the fourth column of the matrix and write

$$\frac{h}{e^2} G^{-1} \begin{pmatrix} I \\ -I \\ 0 \end{pmatrix} = \begin{pmatrix} V_1 \\ V_2 \\ V_3 \end{pmatrix}, \tag{S8}$$

with

$$G = \begin{pmatrix} \sum_{p=1}^3 T_i & -T_1 & -T_3 \\ -T_2 & \sum_{p=1}^3 T_i & -T_1 \\ -T_3 & -T_2 & \sum_{p=1}^3 T_i \end{pmatrix}. \tag{S9}$$

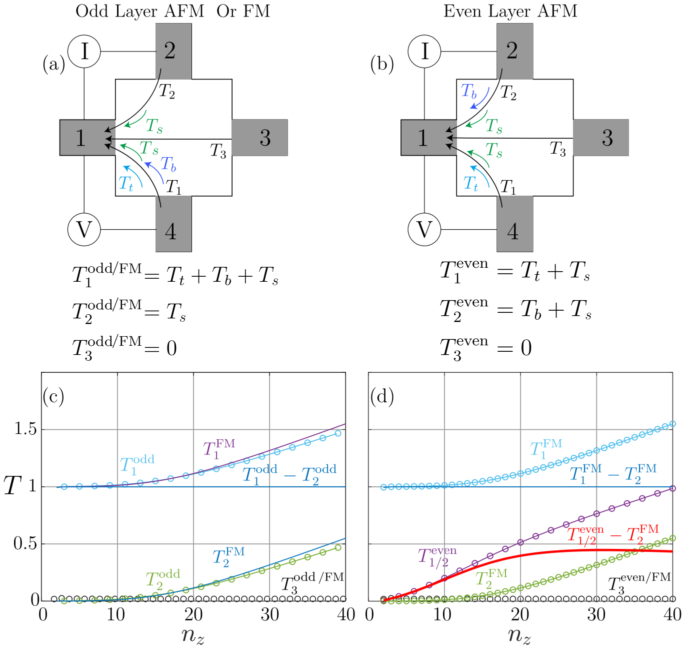

Fig. S12. Schematic of the four-terminal nonlocal measurement in absence of an applied electric field for (a) odd-layer AFM and odd- or even-layer FM topological insulators and (b) the even-layer AFM topological insulator. Different from the surface measurement [S19], here the electrodes (grey) are attached to the side surfaces to measure the transmission coefficients contributed by the top (T_t), bottom (T_b), and side surfaces (T_s). T_1, T_2, and T_3 are the transmission coefficients between two clockwise neighboring, counter-clockwise neighboring, and non-neighboring terminals, respectively. The superscripts "odd", "even", and "FM" are to distinguish the odd-layer AFM, even-layer AFM, and odd- or even-layer FM phases, respectively. (c-d) The numerically calculated transmission coefficients as functions of the thickness (in terms of the number of layers n_z). We take $E_F = 0.01$, $M_0 = 0.4$, $m = 0.12$ and $V = 0$ with the system size $n_x = n_y = 30$.

Therefore, the resistances are given by

$$R_{12} = \frac{V_2 - V_1}{I}, R_{13} = \frac{V_3 - V_1}{I}, R_{14} = \frac{V_4 - V_1}{I}, \tag{S10}$$

In experiments, from the measured nonlocal resistances, one can obtain

$$\begin{aligned}
T_1 &= \frac{h}{e^2} \frac{R_{12}\left(R_{13}+R_{14}\right) - R_{12}^2 - R_{13}^2}{\left[R_{13}^2 + \left(R_{12}-R_{14}\right)^2\right]\left(R_{12}-R_{13}+R_{14}\right)}, \\
T_2 &= \frac{h}{e^2} \frac{R_{12}\left(R_{14}-R_{13}\right) - R_{14}^2}{\left[R_{13}^2 + \left(R_{12}-R_{14}\right)^2\right]\left(R_{12}-R_{13}+R_{14}\right)}, \\
T_3 &= \frac{h}{e^2} \frac{R_{13}^2 - \left(R_{12}+R_{13}\right)R_{14} + R_{14}^2}{\left[R_{13}^2 + \left(R_{12}-R_{14}\right)^2\right]\left(R_{12}-R_{13}+R_{14}\right)}.
\end{aligned} \tag{S11}$$

We first consider the odd-layer AFM topological insulator films, i.e., the Chern insulators. For the ideal case, the system is characterized by one chiral current with $T_1^{\text{odd}} = 1$ and $T_2^{\text{odd}} = T_3^{\text{odd}} = 0$, where the superscript "odd" stands for the odd-layer AFM phase. The numerical results show that the ideal case appears only for ultrathin films with $n_z \leq 5$ [Fig. S12(c)]. Both T_1^{odd} and T_2^{odd} increase with increasing n_z, due to the contribution from the side surfaces. As shown in Figs. S12(a-b), the side-surface transmission coefficient T_s contributes to both clockwise and counter-clockwise directions. Therefore, we have $T_1^{\text{odd}} = T_t + T_b + T_s$ and $T_2^{\text{odd}} = T_s$, where $T_t = T_b = 1/2$ being the contributions from the top and bottom surface layers. As shown by the blue line in Fig. S12(c), it is the difference between the clockwise and counter-clockwise channels (i.e., $T_1^{\text{odd}} - T_2^{\text{odd}}$) that characterizes the Chern-insulator phase in odd-layer AFM topological insulator films.

For even-layer films, when the side surfaces are considered, we have $T_1^{\text{even}} = T_t + T_s$ and $T_2^{\text{even}} = T_b + T_s$, where the superscript "even" stands for the even-layer AFM phase. Due to the $\mathcal{PT}$ symmetry, we have $T_t \equiv T_b$ and

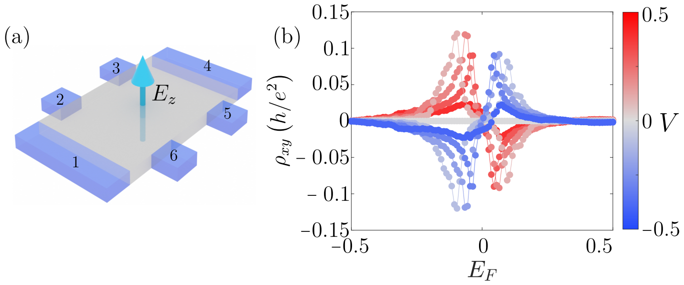

Fig. S13. (a) Schematic of the six-terminal Hall-bar measurement of the AFM insulator. Grey and purple correspond to the AFM insulator and Hall-bar electrodes, respectively. The cyan arrow denotes the perpendicular electric field E_z (modeled by the potential different V between the top and bottom surfaces). (b) Hall resistance ρ_{xy} as a function of E_F for different V (distinguished by the color). We take $M_0 = 0.4$ and $m = 0.12$ with the system size $n_x = 80$, $n_y = 30$, and $n_z = 2$.

$T_1^{\text{even}} \equiv T_2^{\text{even}}$. This agrees well with the results shown in Fig. S12(d). Due to the side surfaces, $T_{1/2}^{\text{even}}$ increase with increasing film thickness n_z and can even exceed 1. We calculate $T_{t/b} = T_{1/2}^{\text{even}} - T_2^{\text{FM}}$ as a function of n_z. For thick films, $T_{t/b} \approx 1/2$, indicating that the system corresponds to an axion insulator. For thinfilms, the side surfaces are gapped due to the quantum confinement and insulating for the chosen E_F, and meanwhile the layer Hall effect is suppressed due to the coupling between the top and bottom surfaces.

Above, we show that the contributions to the nonlocal transport from the layer Hall effect and the side surfaces have the same form, i.e., both of them can lead to the emergence of the counter-propagating chiral currents. Therefore, we propose a method to extract the layer Hall effect from the side-surface contribution as follows. We can tune between the AFM and FM phases by orienting the layer magnetizations using a magnetic field [Fig. S12(d)]. In the FM phase, we can find the side-surface contribution T_2^{FM}. The layer Hall effect then can be found in the AFM phase as $T_{1/2}^{\text{even}} - T_2^{\text{FM}}$.

B. Hall measurement

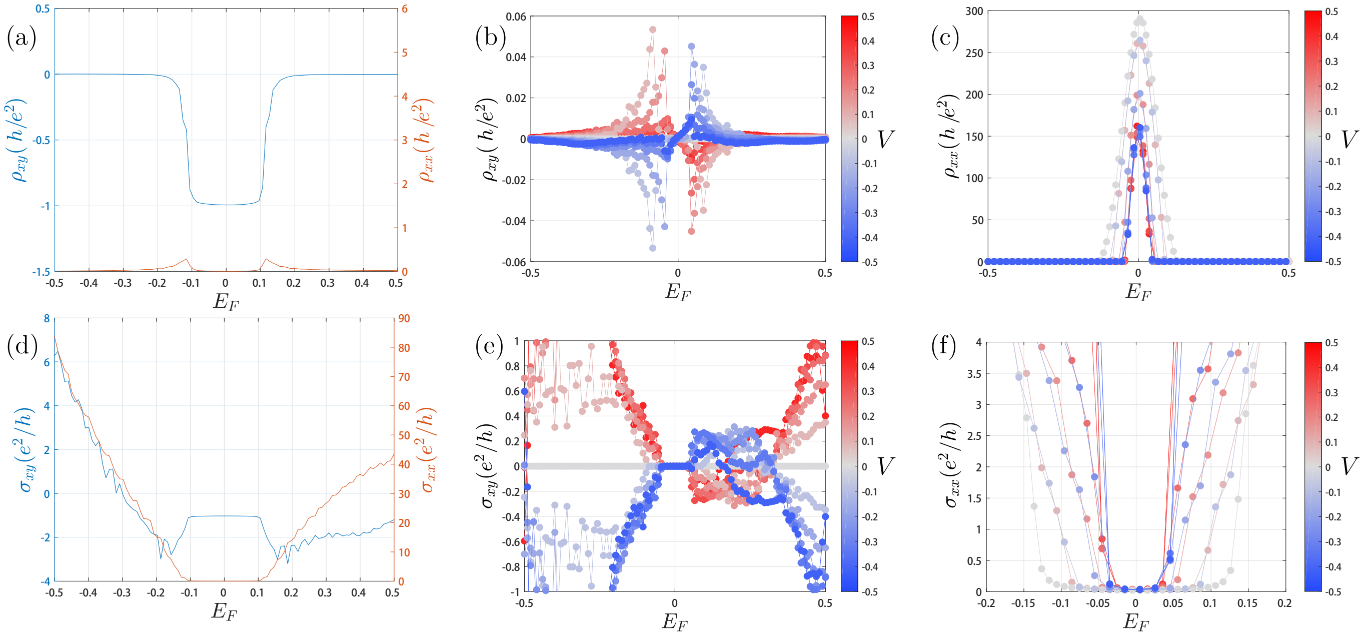

Fig. S14. (a) The Hall resistance ρ_{xy} (blue) and longitudinal resistance ρ_{xx} (red) as functions of E_F. (d) The Hall conductance σ_{xy} (blue) and longitudinal conductance σ_{xx} (red) as functions of E_F. (b) ρ_{xx}, (c) ρ_{xy}, (e) σ_{xy}, and (f) σ_{xx} as functions of E_F. Here, the color distinguishes different V. We take $M_0 = 0.4$ and $m = 0.12$ with the system size $n_x = 80$, $n_y = 30$. The layer thickness is $n_z = 3$ in (a, d), and $n_z = 2$ in (b, c, e, f).

The layer Hall effect can also be experimentally identified by measuring the Hall resistance by applying a perpendicular electric field (modeled by V). We consider a standard six-terminal Hall-bar geometry as depicted in Fig. S13(a). We apply a bias across terminal 1 and terminal 4, and the currents in the voltage probes (terminals 2, 3, 5, and 6) are set to zero. By using the Landauer-Büttiker formula, the voltages V_2, V_3, V_5, and V_6 of the voltage probes and the currents I_1 and I_4 can be calculated. Owing to Kirchoff's law, $I_1 = -I_4 = I$.

We start with a three-layer AFM topological insulator, which corresponds to a Chern insulator. We calculate the transport in a standard six-terminal Hall-bar geometry as depicted in Fig. 7(a) in the main text. From the Landauer-Büttiker formula [S12–S14], the longitudinal and Hall resistances are given by $\rho_{xx} = (V_2 - V_3)/I$ and $\rho_{xy} = (V_2 - V_6)/I$, respectively. As shown in Fig. S14(a), the results are as expected. When the Fermi energy lies inside the band gap, there has a Hall resistance plateau with $\rho_{xy} = 1$ and the corresponding $\rho_{xx} = 0$, indicating a Chern insulator phase. When the Fermi energy lies outside the band gap, both ρ_{xy} and ρ_{xx} drop to zero.

Equivalently, we have

$$\sigma_{xy} = \rho_{yx}/\left(\rho_{yx}^2 + \rho_{xx}^2\right),$$
$$\sigma_{xx} = \rho_{xx}/\left(\rho_{yx}^2 + \rho_{xx}^2\right), \tag{S12}$$

and the results are shown in Fig. S14(d). Inside the band gap, the result of σ_{xy} is consistent with the result obtained by the Kubo formula [Fig. S3(a)], except the deviation when the Fermi energy lies outside the band gap. The deviation is attributed to the metallic bulk states in the Landauer-Büttiker formulism, as revealed in the previous studies [S20].

For the two-layer AFM topological insulator, σ_{xy} is zero when the Fermi energy is located inside the band gap [Fig. S14(e)]. For Fermi energies slightly larger than zero, there appears a Hall conductance plateau with its sign switchable by the direction the perpendicular electric field. Further increasing E_F, the Hall conductance deviates from the results obtained from the Kubo formula, because the metallic bulk states dominate the transport. This is similar to the case of the three-layer AFM topological insulator shown in Fig. S14(b).

On the other hand, the numerical results of σ_{xy} and σ_{xx} are very sensitive to a metallic phase with $\rho_{xy} \to 0$ and $\rho_{xx} \to 0$. For example, by varying the chemical potential of the electrodes, the mismatch between the electrodes and the device can induce minor changes in ρ_{xy} and ρ_{xx}, but lead to significant changes in σ_{xy} and σ_{xx} according to Eq. (S12). This is also observed in Fig. S14(e), σ_{xy} shows a significant difference between $E_F > 0$ and $E_F < 0$, which is attributed to the mismatch between the electrodes and the device.

Figure S13(b) shows the numerically calculated Hall resistances as functions of E_F. For a certain $V > 0$, the Hall resistance R_H has two peaks with opposite signs for $E_F > 0$ and $E_F < 0$, indicating the emergence of the layer Hall effect. More interestingly, the Hall plateaus reverse their signs when the electric field direction is reversed. Therefore, we propose that the layer Hall effect could be macroscopically revealed in the Hall measurement by applying a perpendicular electric field.

Our simulation shows only a nearly half-quantized Hall conductance in the proposed candidates of the axion insulator, which could be even harder to be realized in experiments. Specifically, when the Fermi energy lies in the gap of one surface (insulating) but crosses an energy band on the other surface (metallic), the Hall conductance is nearly half-quantized on the insulating surface but nearly zero on the metallic surface. Therefore, the half-quantized Hall currents may be shorted by the metallic top or bottom surface. Nevertheless, the layer Hall effect is significantly enhanced in the possible candidates of the axion insulator.

Furthermore, in a very recent work, the half-quantized Hall conductance has been observed in a semi-magnetic topological insulator, where the top surface is gapped by magnetic doping and the bottom surface is non-magnetic and gapless [S21], which shares a similar scenario of the measurement of the layer Hall effect in a perpendicular electric field. A theory suggests that disorder can localize the metallic states, so the quantization of the edge states can survive [S22]. Therefore, we expect that the half-quantization in the layer Hall effect could be experimentally observed in the future.

Above, we focus on the case that the side surface states are gapless Dirac cone protected by the combined antiferromagnetic time-reversal symmetry. We argue that our proposals also work for arbitrary side surface states. In the first measurement, the contributions to the nonlocal transport from any side surface (gapped or gapless) can be obtained by measuring the nonlocal resistances between properly chosen electrodes in the FM phase. By comparing the results between the FM and AFM phases, the contributions from the top and bottom surfaces can be extracted and then the layer Hall effect can be identified. In the second measurement, the Hall measurement is independent on the side surfaces, because they contribute a zero net Hall conductance due to their fully compensated magnetization.

SV. DFT CALCULATIONS

It was noticed that AFM $MnSb_2Te_4$ is located near the boundary of a topological phase transition, and whether it is a topological insulator or a trivial insulator highly depends on the in-plane lattice parameter [S23–S25]. This

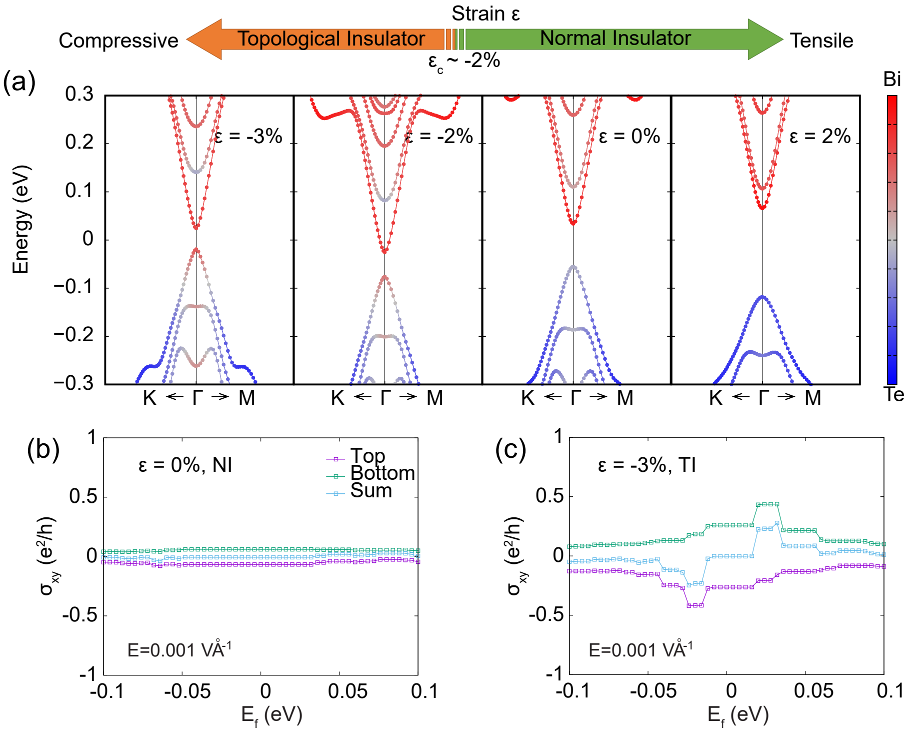

Fig. S15. Strain engineering of topological phase transition in $MnSb_2Te_4$ and the layer Hall effect. (a) Band structures of a four-layer $MnSb_2Te_4$ slab under different uniaxial strain along the z-direction. The upper panel is an illustration of the strain-induced topological phase transition. [(b) and (c)] The Hall conductance for the top and bottom surfaces as well as their sum for the four-layer $MnSb_2Te_4$ at different values of strain. The applied electric field is 1 mV/Å.

provides us an ideal platform to examine if the band topology can induce quantitatively distinct σ_{xy} responses. We applied the strain along the z-direction with the range (-5%, 5%) covering the topological phase transition. We find that a compressive strain at -2% can lead to a band inversion between the Bi and Te states [Fig. S15(a)]. Distinct layer Hall conductance responses across the topological phase transition are observed: Under the applied electric field of 1 mV/Å, the TI phase (e.g., strain $\varepsilon = -3\%$) yields $\sigma_{xy} \sim 0.44\ e^2/h$ for the surface layer and $\sim 0.24\ e^2/h$ for their sum, while at the normal insulator phase (e.g., $\varepsilon = 0\%$) these values drop significantly to 0.058 and 0.019 e^2/h, respectively, almost one order smaller, as shown in Figs. S15(b) and S15(c), which clearly distinguish the two phases of $MnSb_2Te_4$. By combining the comparison between $MnBi_2Te_4$ and $MnSb_2Te_4$ and between different phases in $MnSb_2Te_4$, we conclude that the proposed layer Hall effect is highly sensitive to the Berry curvature variation and band inversion. In this sense, the layer Hall effect can be regarded as an efficient experimental signature to identify topological trivial and nontrivial insulators.

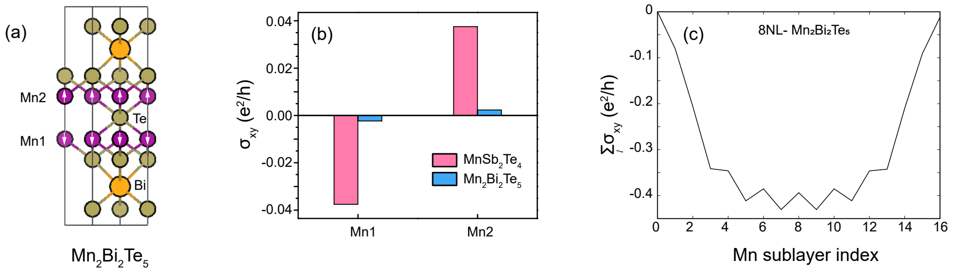

Fig. S16. Crystal structure of $Mn_2Bi_2Te_5$. (b) Layer-resolved Hall conductance for the single layer $Mn_2Bi_2Te_5$ and bilayer $MnSb_2Te_4$, both of which are topological trivial and consist of two Mn-sublayers. (c) Integrated layer Hall conductance for an 8-NL $Mn_2Bi_2Te_5$ slab.

We also performed DFT calculations for two systems without the topological enhancement, the bilayer $MnSb_2Te_4$

and monolayer $Mn_2Bi_2Te_5$. $Mn_2Bi_2Te_5$ is an AFM TI [S26–S28] consisting of nine atomic layers (nonuple layers, NLs) within each unit, including two Mns with opposite spin alignments bridged by the center Te atom, as shown in Fig. S16(a). The comparison between these two systems plotted in Fig. S16(b) shows that the non-vdW-connected $\mathcal{PT}$-partners in $Mn_2Bi_2Te_5$ indeed manifest a smaller layer Hall effect than the vdW-connected system $MnSb_2Te_4$. However, we emphasize here that this is a material-specific result. The detailed electronic band structure and material chemistry should play a key role in determining the layer Hall effect.

Furthermore, we calculated the topological enhancement in $Mn_2Bi_2Te_5$. When the thickness of the $Mn_2Bi_2Te_5$ slab is increased, the local hidden Berry curvature, and hence the layer-resolved Hall conductance, increase as the thickness of the slab increases. In Fig. S16(c) we show the integrated layer-resolved Hall conductance for an 8-NL $Mn_2Bi_2Te_5$. It is found that the sum of the surface Hall conductance gradually accumulates and reaches to near 0.43 e^2/h at the center of the slab, close to that observed in $MnBi_2Te_4$ [S29]. After applying a normal electric field of 0.1 meV/Å to this 8-NL slab, we obtained the layer Hall effect $\sigma_{xy}^{\mathrm{LHE}} = 0.27e^2/h$ when the Fermi energy is raised by 10 meV, a similar order of magnitude to that of $MnBi_2Te_4$.

[S1] D. Zhang, M. Shi, T. Zhu, D. Xing, H. Zhang, and J. Wang, "Topological axion states in the magnetic insulator $MnBi_2Te_4$ with the quantized magnetoelectric effect", Phys. Rev. Lett. **122**, 206401 (2019).

[S2] R.-X. Zhang, F. Wu, and S. Das Sarma, "Möbius insulator and higher-order topology in $MnBi_{2n}Te_{3n+1}$", Phys. Rev. Lett. **124**, 136407 (2020).

[S3] H. Li, H. Jiang, C.-Z. Chen, and X. C. Xie, "Critical behavior and universal signature of an axion insulator state", Phys. Rev. Lett. **126**, 156601 (2021).

[S4] C.-Z. Chen, H. Liu, and X. C. Xie, "Effects of Random Domains on the Zero Hall Plateau in the Quantum Anomalous Hall Effect", Phys. Rev. Lett. **122**, 026601 (2019).

[S5] E. Prodan, "Robustness of the spin-Chern number", Phys. Rev. B **80**, 125327 (2009).

[S6] E. Prodan, "Disordered topological insulators: a non-commutative geometry perspective", J. Phys. A: Math. Theor. **44**, 113001 (2011).

[S7] C.-Z. Chen, J. Song, H. Jiang, Q.-f. Sun, Z. Wang, and X. C. Xie, "Disorder and metal-insulator transitions in Weyl semimetals", Phys. Rev. Lett. **115**, 246603 (2015).

[S8] R. Chen, C.-Z. Chen, J.-H. Sun, B. Zhou, and D.-H. Xu, "Phase diagrams of Weyl semimetals with competing intraorbital and interorbital disorders", Phys. Rev. B **97**, 235109 (2018).

[S9] R. Bianco and R. Resta, "Mapping topological order in coordinate space", Phys. Rev. B **84**, 241106(R) (2011).

[S10] M. D. Caio, G. Moller, N. R. Cooper, and M. J. Bhaseen, "Topological marker currents in chern insulators", Nature Physics **15**, 257 (2019).

[S11] N. Varnava and D. Vanderbilt, "Surfaces of axion insulators", Phys. Rev. B **98**, 245117 (2018).

[S12] R. Landauer, "Electrical resistance of disordered one-dimensional lattices", Philos. Mag. **21**, 863 (1970).

[S13] M. Büttiker, "Absence of backscattering in the quantum Hall effect in multiprobe conductors", Phys. Rev. B **38**, 9375 (1988).

[S14] D. S. Fisher and P. A. Lee, "Relation between conductivity and transmission matrix", Phys. Rev. B **23**, 6851 (1981).

[S15] A. MacKinnon, "The calculation of transport properties and density of states of disordered solids", Z. Phys. B **59**, 385 (1985).

[S16] G. Metalidis and P. Bruno, "Green's function technique for studying electron flow in two-dimensional mesoscopic samples", Phys. Rev. B **72**, 235304 (2005).

[S17] A. Roth, C. Brune, H. Buhmann, L. W. Molenkamp, J. Maciejko, X.-L. Qi, and S.-C. Zhang, "Nonlocal transport in the quantum spin Hall state", Science **325**, 294 (2009).

[S18] Y. Li, C. Liu, Y. Wang, Z. Lian, H. Li, Y. Wu, J. Zhang, and Y. Wang, "Nonlocal Transport in Axion Insulator State of $MnBi_2Te_4$", arXiv:2105.10390 (2021).

[S19] R.-L. Chu, J. Shi, and S.-Q. Shen, "Surface edge state and half-quantized Hall conductance in topological insulators", Phys. Rev. B **84**, 085312 (2011).

[S20] S. guang Cheng, "The quantum anomalous hall effect in a topological insulator thin film —the role of magnetic disorder", EPL (Europhysics Letters) **105**, 57004 (2014).

[S21] M. Mogi, Y. Okamura, M. Kawamura, R. Yoshimi, K. Yasuda, A. Tsukazaki, *et al.*, "Experimental signature of the parity anomaly in a semi-magnetic topological insulator", Nature Physics **18**, 390 (2022).

[S22] Y.-Y. Zhang, M. Shen, X.-T. An, Q.-F. Sun, X.-C. Xie, K. Chang, and S.-S. Li, "Coexistence and decoupling of bulk and edge states in disordered two-dimensional topological insulators", Phys. Rev. B **90**, 054205 (2014).

[S23] L. Zhou, Z. Tan, D. Yan, Z. Fang, Y. Shi, and H. Weng, "Topological phase transition in the layered magnetic compound $MnSb_2Te_4$: Spin-orbit coupling and interlayer coupling dependence", Phys. Rev. B **102**, 085114 (2020).

[S24] H. Zhang, W. Yang, Y. Wang, and X. Xu, "Tunable topological states in layered magnetic materials of $MnSb_2Te_4$, $MnBi_2Se_4$, and $MnSb_2Se_4$", Phys. Rev. B **103**, 094433 (2021).

[S25] S. V. Eremeev, I. P. Rusinov, Y. M. Koroteev, A. Y. Vyazovskaya, M. Hoffmann, P. M. Echenique, A. Ernst, M. M. Otrokov, and E. V. Chulkov, "Topological Magnetic Materials of the $(MnSb_2Te_4)(Sb_2Te_3)_n$ van der Waals Compounds

Family", J. Phys. Chem. Lett. **12**, 4268 (2021).
[S26] J. Zhang, D. Wang, M. Shi, T. Zhu, H. Zhang, and J. Wang, "Large Dynamical Axion Field in Topological Antiferromagnetic Insulator $Mn_2Bi_2Te_5$", Chin. Phys. Lett. **37**, 077304 (2020).
[S27] Y. Li, Y. Jiang, J. Zhang, Z. Liu, Z. Yang, and J. Wang, "Intrinsic topological phases in $Mn_2Bi_2Te_5$ tuned by the layer magnetization", Phys. Rev. B **102**, 121107 (2020).
[S28] L. Cao, S. Han, Y.-Y. Lv, D. Wang, Y.-C. Luo, Y.-Y. Zhang, *et al.*, "Growth and characterization of the dynamical axion insulator candidate $Mn_2Bi_2Te_5$ with intrinsic antiferromagnetism", Phys. Rev. B **104**, 054421 (2021).
[S29] M. Gu, J. Li, H. Sun, Y. Zhao, C. Liu, J. Liu, H. Lu, and Q. Liu, "Spectral signatures of the surface anomalous Hall effect in magnetic axion insulators", Nature Commun. **12**, 3524 (2021).
